# Time-limited deferral scenarios and blood donation intentions among men who have sex with men in China: a nationwide mixed-methods study

**DOI:** 10.1186/s40249-026-01496-9

**Published:** 2026-09-10

**Authors:** Guozhen Wu, Xinsheng Wu, Thomas S. Fitzpatrick, Lin He, Yong Lu, Yuecheng Yang, Chengliang Chai, Lin Ouyang, Lijuan Wang, Haibo Jiang, Hui Wang, Lijing Wang, Ping Ma, Xiao Qi, Xiaojun Meng, Song Fan, Yingjie Liu, Huachun Zou

**Affiliations:** 1 Shanghai Institute of Infectious Disease and Biosecurity, School of Public Health, Fudan University, 130 Dongan Road, Xuhui District, Shanghai, 200032 People’s Republic of China; 2 Center for AIDS Research (CFAR), School of Public Health, Key Laboratory of Public Health Safety, Ministry of Education, Fudan University, Shanghai, China; 3 Department of Medicine, University of Washington, Seattle, WA USA; 4 Zhejiang Provincial Center for Disease Control and Prevention, Hangzhou, Zhejiang China; 5 School of Public Health, The Key Laboratory of Environmental Pollution Monitoring and Disease Control, Ministry of Education, Guizhou Medical University, Guiyang, Guizhou China; 6 Dehong Prefecture Center for Disease Control and Prevention, Mangshi, Yunnan China; 7 Chongqing Center for Disease Control and Prevention, Chongqing, China; 8 Chaoyang District Center for Disease Control and Prevention, Beijing, China; 9 Ningbo Center for Disease Control and Prevention, Ningbo, Zhejiang China; 10 Haishu District Center for Disease Control and Prevention, Ningbo, Zhejiang China; 11 Shijiazhuang Fifth Hospital, Shijiazhuang, Hebei China; 12 The Second Affiliated Hospital of Tianjin Medical University, Tianjin, China; 13 Wuxi Center for Disease Control and Prevention, Wuxi, Jiangsu China; 14 School of Public Health, Southwest Medical University, Luzhou, Sichuan China; 15 Faculty of Medicine Nursing and Health Sciences, Melbourne Sexual Health Centre, Monash University, Melbourne, Australia

**Keywords:** Men who have sex with men (MSM), Blood donation, Deferral, Human immunodeficiency virus (HIV), China

## Abstract

**Background:**

Approaches to managing blood donation eligibility for men who have sex with men (MSM) vary across countries, and time-limited deferral policies have been adopted in an increasing number of jurisdictions. Our study aimed to evaluate attitudes towards and acceptance of time-limited blood donation deferral periods among MSM in China.

**Methods:**

We used an explanatory sequential mixed-methods design, consisting of a nationwide cross-sectional survey of MSM in China from April to July 2024, followed by qualitative interviews. The survey collected sociodemographic characteristics, sexual behaviors, blood donation history, and attitudes towards four hypothetical deferral scenarios (12-, 6-, 3-, and 1-month deferral after most recent same-sex sexual behavior). We assessed willingness to donate under each scenario and estimated scenario-specific behavioral eligibility based on self-reported anal sex with another man during the corresponding lookback period. Multivariable modified Poisson regression, adjusted for age, education, and income, was used to identify factors associated with support for hypothetical time-limited deferral policies. Semi-structured interviews explored perceptions of the current prohibition and hypothetical deferral approaches and were used to explain and contextualize the survey findings.

**Results:**

A total of 2725 MSM participated. Overall, 48% were neutral and 32% were supportive of hypothetical time-limited deferral approaches. As the hypothetical deferral period shortened from 12 months to 1 month, willingness to donate increased among both previous donors (44%, 50%, 61%, 74%; *P* for trend < 0.001) and non-donors (26%, 27%, 33%, 45%; *P* for trend < 0.001). Among MSM willing to donate under each scenario, the proportions reporting no anal sex with another man during the corresponding periods were 37%, 42%, 45%, and 53%, respectively. In qualitative interviews (*n* = 16), most participants (12/16) viewed hypothetical deferral approaches as more acceptable than the current lifelong prohibition.

**Conclusions:**

Many MSM in China expressed willingness to donate blood under hypothetical time-limited deferral scenarios, with higher acceptance under shorter deferral periods. These findings provide empirical evidence for future evaluation of whether shorter time-limited deferral approaches could improve acceptability and intended compliance while maintaining blood safety within China’s existing donor screening and surveillance system.

**Supplementary Information:**

The online version contains supplementary material available at https://doi.org/10.1186/s40249-026-01496-9.

## Background

Blood donation eligibility criteria for men who have sex with men (MSM) have evolved significantly over the past three decades in many countries. In the early stages of the human immunodeficiency virus (HIV) epidemic, transfusion-related transmission resulted in numerous infections, and MSM were identified as a high-risk population for HIV [1]. To mitigate HIV transmission risks and ensure blood safety, in the 1980 s many countries implemented prohibition against blood donation from MSM [2–4]. In China, the emergence of illegal commercial blood collection during the 1990 s contributed to HIV transmission [5]. In response, the government introduced regulatory measures, including the *Whole Blood and Component Donor Selection Requirements*, which established a lifelong prohibition on blood donation from MSM [6].

Over the past decade, many countries have shifted from lifelong prohibition to time-limited deferral policies for MSM. Time-limited deferral policies refer to donor eligibility criteria that temporarily defer MSM from blood donation for a specified period after their most recent male-to-male sexual behavior, rather than permanently excluding them from donation [1, 7, 8]. The rationale for such policies is closely related to the HIV testing window period, defined as the interval between HIV acquisition and the point at which infection can be reliably detected by available screening tests [9]. During this period, a recently infected donor may have a non-reactive screening result despite having acquired HIV. Deferral periods are therefore intended to provide a behavioral lookback interval that reduces the likelihood of donation during a period of undetectable recent infection, while allowing donation after the deferral period has elapsed. Deferral periods vary across jurisdictions and commonly range from several months to 1 year [1, 7, 8]. New Zealand adopted a 12-month deferral policy in 2014 [2]. The United States switched to a 3-month deferral period in 2020 [10, 11]. Australia implemented a 3-month deferral policy in 2021 [4, 12]. Several countries, including France and Canada, have transitioned to gender-neutral and individualized risk-based screening approaches [13, 14].

This global shift has been influenced by several key factors: (1) advancements in HIV testing technology, particularly modern nucleic acid testing, have significantly shortened the detection window for acute HIV infection to under 10 days, with high sensitivity and specificity [7, 9]. (2) Risk of transfusion-transmitted HIV has declined substantially [15]. In the United States, the current risk of acquiring HIV from a transfusion is approximately 0.5 cases per million transfusion [16], and the National Health Commission of China reported virtually no transfusion-transmitted HIV infections from 2016 to 2020 [17]. (3) Blood shortages have prompted policymakers to revise policies to increase blood donation. The United States and Brazil faced blood supply crises during the COVID-19 pandemic, leading to the implementation of time-limited deferral periods for MSM who wanted to donate blood [18, 19]. (4) Concerns about proportionality and perceived discrimination have also contributed to policy debate. In Israel, community activists criticized the 12-month deferral period, leading to the adoption of the Frozen Plasma Quarantine Programme (FPQP) in 2018 and a transition to a gender-neutral policy in 2021 [8].

China faces ongoing pressure to maintain an adequate and safe blood supply [20]. In this context, locally relevant evidence is needed to evaluate how blood donation eligibility criteria can maintain blood safety while also being acceptable, understandable, and feasible to implement in practice. Under the current MSM blood donation eligibility criteria in China, implementation relies partly on donor self-disclosure of sexual behaviors during pre-donation screening. Real-world adherence may therefore be incomplete, and previous studies have suggested that some MSM have donated blood despite the current prohibition. In a 2018 study, 29% of MSM in Shenzhen reported having ever donated blood [21]. These findings suggest that, beyond the existence of eligibility criteria, it is also important to understand how MSM interpret these criteria, whether they intend to adhere to them, and whether more behavior-specific approaches could identify individuals who are both willing to donate and meet defined behavioral criteria. However, existing evidence from China remains limited regarding how MSM perceive alternative blood donation eligibility approaches and whether they would meet behavioral eligibility requirements under such approaches. To address these gaps, our study assessed attitudes towards the current prohibition and hypothetical time-limited deferral approaches among MSM in China, estimated the prevalence of prior blood donation, described past donation experiences, and evaluated willingness to donate and scenario-specific behavioral eligibility under different hypothetical deferral periods.

## Methods

### Study design

We used an explanatory sequential mixed-methods design, consisting of a nationwide multicenter cross-sectional survey followed by qualitative interviews from April to July 2024. The quantitative phase assessed blood donation experiences and responses to current and hypothetical MSM blood donation eligibility criteria. The qualitative phase used semi-structured interviews with a purposively selected subset of survey respondents to explain and contextualize the survey findings.

To ensure geographic representation, we selected study sites across seven regions: Beijing, Tianjin and Hebei in north China; Guangdong and Guangxi in south China; Shandong, Anhui, Zhejiang and Jiangsu in east China; Henan, Hunan and Hubei in central China; Liaoning and Jilin in northeast China; Xinjiang in northwest China; and Sichuan, Chongqing, Guizhou and Yunnan in southwest China. At each site, we partnered with Centers for Disease Prevention and Control (CDC), infectious disease hospitals, and MSM community health centers to recruit participants. Survey eligibility criteria were defined as being male, aged 18–55 years (legal age range for blood donation in China) and having ever had either oral or anal sex with another man or identifying as gay or bisexual. Based on a previous study reporting a 29% blood donation rate among MSM [21], and assuming a 95% confidence level, a 2% margin of error, and an anticipated 80% of valid responses, the minimum required sample size to estimate the prevalence of blood donation among MSM was 2473 participants.

### Quantitative recruitment and data collection

The survey instrument was developed by the research team and hosted on *Wenjuanxing* (Changsha Ranxing Information Technology Co., Ltd., Changsha, China; https://www.wjx.cn), a Chinese online survey platform. Before formal data collection, we convened a meeting with coordinators from all participating sites. 16 site coordinators first completed the survey themselves and then pilot-tested it among 51 MSM participants at their sites to assess clarity, acceptability, and completion burden. Based on feedback from site coordinators and pilot participants, we revised the survey by reducing the number of items and refining the wording of several questions.

After the survey was finalized, the survey link was distributed to each participating site. Recruitment was conducted through both online and in-person approaches. For online recruitment, local site coordinators and MSM community organizations distributed the survey link or QR code through online MSM community networks and chat groups. For in-person recruitment, staff at collaborating Centers for Disease Control and Prevention, infectious disease hospitals, and MSM community health centers approached eligible MSM when they attended HIV testing or other community-based health services and invited them to complete the web-based survey by scanning the QR code or opening the survey link on their own mobile devices. Regardless of recruitment approach, all participants completed the same self-administered electronic survey, and responses were uploaded directly to *Wenjuanxing*.

Before completing the survey, participants were provided with an informed consent form describing the study purpose, survey content, anonymity, information protection, voluntary participation, reimbursement, and an approximate survey duration of 15 min. The required survey items were anonymous. Participants who were willing to be contacted for a subsequent qualitative interview could voluntarily provide a telephone number, which was used only for interview recruitment. Survey respondents received a reimbursement of CNY 30 (USD 4.1). During data collection, we reviewed submitted surveys and excluded invalid responses according to predefined quality-control criteria, including age ineligibility, duplicate submissions, incorrect responses to quality-control questions, and clearly invalid response patterns, such as selecting the same response option for all questions.

### Survey measures

The survey collected data across several domains. (1) Sociodemographic characteristics included age, income, educational level, sexual orientation, city of residence, lifetime recreational drug use, and disclosure of sexual orientation to friends, family, coworkers, or healthcare providers. (2) Questions assessed history of sex with women and oral or anal sex with men in the past 12, 6, 3, and 1 months, as well as number of sex partners, sexualized drug use and condom use during insertive sex. To approximate behavioral deferral criteria used in current international blood donor eligibility guidelines, we focused on anal sex with men during the relevant time window for each scenario as the primary marker of recent higher-risk sexual exposure [22, 23]. (3) Blood donation history included frequency of donation, time of most recent donation, and motivation for donation. Respondents were classified as donors or non-donors based on donation history. (4) Attitudes towards blood donation eligibility criteria measured both attitudes towards the current blanket prohibition and hypothetical alternative time-limited deferral policies. Respondents were asked whether they supported, opposed, or felt neutral about 12-, 6-, 3-, and 1-month deferral policies. Because the concept of a deferral policy is unfamiliar in China, a brief explanation of the policy was provided before this set of questions, and a fourth response option (“still unclear about the meaning of the deferral policy”) was included to capture uncertainty. For each hypothetical deferral period, respondents were asked two binary yes/no questions: whether they would plan to donate blood in the next 5 years if eligible under that deferral period, and whether they would comply with abstaining from sex with another man before donating blood for each hypothetical deferral period. If respondents reported they were willing and planned to donate under a hypothetical deferral period, they were asked the planned frequency of donations and the amount per donation.

### Quantitative analysis

Descriptive statistics were used to summarize sociodemographic information, sexual behaviors, previous blood donation behaviors, and attitudes, acceptance, and willingness to donate blood under the four hypothetical deferral periods. Univariate logistic regression tests were used for univariate analysis to identify correlates. We used multivariable modified Poisson regression with robust variance to examine correlates of support for hypothetical time-limited deferral policies. Attitudes towards the hypothetical deferral policy were dichotomized for the primary analysis as support versus non-support (neutral/oppose) to facilitate interpretation. We evaluated potential correlates including sociodemographic characteristics, sexual behaviors assessed over the timeframe corresponding to each hypothetical deferral period, and attitudes towards blood donation and the current prohibition. For each potential correlate, we estimated prevalence ratios and age-, education-, and income-adjusted prevalence ratios. Multicollinearity among covariates was assessed using generalized variance inflation factors (GVIFs). For categorical variables with more than two levels, GVIFs were adjusted as ${\mathrm{G}\mathrm{V}\mathrm{I}\mathrm{F}}^{\frac{1}{2\times \mathrm{d}f}}$. The maximum adjusted GVIF was 1.13, indicating no evidence of problematic multicollinearity. Results from the multivariate modified Poisson regression models were reported as adjusted prevalence ratios (aPR) with 95% confidence intervals (*CI*). A two-tailed *P* < 0.05 was considered statistically significant. To evaluate trends in willingness to donate across the ordered hypothetical deferral scenarios, we used generalized estimating equations (GEE) with a logit link, binomial distribution, and participant ID as the clustering variable. Deferral scenario was coded as an ordinal variable, with higher values indicating shorter deferral periods from the 12-month to the 1-month scenario. An odds ratio greater than 1 indicated higher willingness to donate under shorter hypothetical deferral periods. All analyses were performed using R software version 4.4.2 (R Foundation for Statistical Computing, Vienna, Austria).

### Qualitative data collection

At the end of the quantitative survey, respondents were asked whether they would be willing to participate in a follow-up qualitative interview. Those who agreed could voluntarily provide a telephone number for interview recruitment. After preliminary analysis of the quantitative survey data, we developed a semi-structured interview guide focusing on key issues that required further explanation, including perceptions of the current prohibition on donation, attitudes towards hypothetical time-limited deferral policies, preferred deferral period length, perceived feasibility of complying with deferral requirements, and willingness to donate under alternative eligibility scenarios. The interview guide was informed by the Theory of Planned Behavior [24], which highlights the influence of behavioral attitudes, subjective norms, and perceived behavioral control on actual behavior.

Before each telephone interview, the interviewer reconfirmed informed consent and obtained permission for audio recording. Interviews lasted approximately 30 min. Interview respondents received a reimbursement of CNY 100 (USD 13.9). Recruitment continued until thematic saturation was reached. Recruitment and preliminary analysis were conducted iteratively: after each interview, the research team reviewed the transcript or interview summary and assessed whether new themes, codes, or explanations relevant to the quantitative findings had emerged. We considered thematic saturation to have been reached when additional interviews no longer generated new themes or substantially new explanations related to perceptions of the current eligibility criteria, hypothetical time-limited deferral policies, perceived feasibility, or willingness to donate.

### Qualitative analysis

Interviews were conducted via telephone and audio recorded. Each recorded interview was transcribed verbatim. We removed repeated filler phrases, clarified pronoun references, corrected obvious transcription errors. Qualitative data were analyzed using NVivo11 software (QSR International Pty Ltd., Melbourne, Australia).

We used a combination of deductive and inductive thematic analysis. An initial codebook was developed based on the interview guide and the main topics of interest, including blood donation experiences, motivations for donation, awareness of current eligibility criteria, attitudes towards the current prohibition, and perceptions of hypothetical time-limited deferral policies. Interview transcripts were then coded in NVivo. During coding, additional codes were added inductively when new ideas emerged from the transcripts. The codebook was refined iteratively as coding progressed, and related codes were grouped to develop broader themes.

To enhance the rigor of the qualitative analysis, two researchers independently reviewed interview transcripts and coding outputs to assess whether the codebook captured both predefined topics and newly emerging ideas. One researcher (GW) checked coded transcripts against the codebook to evaluate the completeness and consistency of data extraction, while another researcher (XW) revisited selected audio recordings to verify interpretive accuracy. Divergent interpretations were discussed until consensus was achieved. We additionally presented preliminary interpretations to site coordinators, whose feedback contributed to refining the final thematic synthesis.

### Mixed-methods synthesis

Qualitative and quantitative findings were integrated through side-by-side comparison to identify concordance, discordance, or expansion between survey and interview results and to generate meta-inferences. Concordant results reflected mutual reinforcement, discordant results indicated inconsistency, and expansion captured complementary insights. Interview quotes were selected, translated into English, and cross-verified by two bilingual researchers.

## Results

**Fig. 1:**
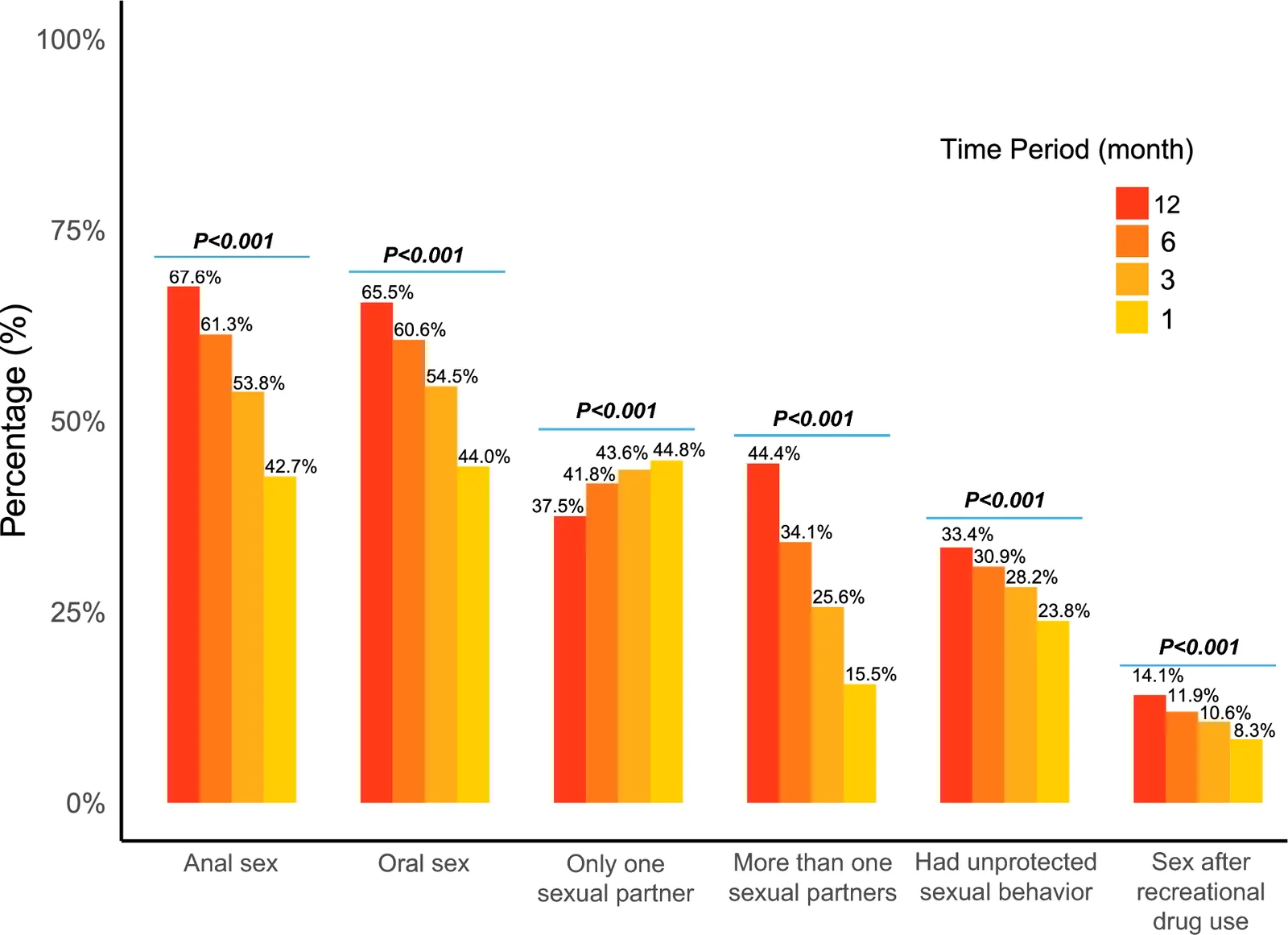
Sexual behavior patterns of men who have sex with men over different time periods in the past

A total of 3222 surveys were collected, of whom 497 were excluded as invalid responses based on survey eligibility criteria (389) and quality-control checks (108). The 2725 (85%) valid responses were included in the quantitative analysis. Survey respondents had a median age of 28 years (IQR: 23–35), 42% had attended college, 54% had monthly income below 5000 CNY (USD 689.3), and 42% had disclosed their sexual orientation to friends, family, coworkers, or healthcare providers (Table 1). Figure 1 illustrates the sexual behaviors of MSM in the 12-, 6-, 3-, and 1-month periods prior to survey completion. With each successively shorter period, the proportions reporting anal sex, oral sex, having more than one sexual partner, condomless insertive sex, and sex after recreational drug use decreased.

**Table 1:** Sociodemographic characteristics and HIV testing history among men who have sex with men in China stratified by blood donation history

	Total (*N* = 2725)	Donor (*N* = 887)	Non-donor (*N* = 1838)
	*n* (%)	*n* (%)	*n* (%)
Sociodemographic characteristics			
Age, median (IQR), years	28 (IQR: 23–35)	29 (IQR: 25–35)	28 (IQR: 22–35)
Highest educational attainment			
Junior high school and below	190 (7.0)	49 (5.5)	141 (7.7)
Senior high school/technical secondary school	436 (16.0)	110 (12.4)	326 (17.7)
Junior college/associate degree	731 (26.8)	237 (26.7)	494 (26.9)
Bachelor’s degree	1136 (41.7)	390 (44.0)	746 (40.6)
Graduate degree or above	232 (8.5)	101 (11.4)	131 (7.1)
Monthly income, CNY (1 CNY = 0.14 USD)			
No income	474 (17.4)	118 (13.3)	356 (19.3)
< 2000	180 (6.6)	52 (5.9)	128 (7.0)
2000–4999	813 (29.8)	253 (28.5)	560 (30.5)
5000–9999	850 (31.2)	286 (32.2)	564 (30.7)
≥ 10,000	408 (15.0)	178 (20.1)	230 (12.5)
Sexual orientation			
Gay	2078 (76.3)	670 (75.6)	1408 (76.6)
Bisexual	509 (18.7)	164 (18.5)	345 (18.8)
Heterosexual	128 (4.7)	50 (5.6)	78 (4.2)
Other	10 (0.3)	3 (0.3)	7 (0.4)
Disclosure of sexual orientation to friends, family, coworkers, or health-care providers			
Ever	1147 (42.1)	400 (45.1)	747 (40.6)
Never	1578 (57.9)	487 (54.9)	1091 (59.4)
Recreational drug use in lifetime			
Ever	305 (11.2)	109 (12.3)	196 (10.7)
Never	2420 (88.8)	778 (87.7)	1642 (89.3)
Circumcision			
Yes	525 (19.3)	217 (24.5)	308 (16.8)
No	2200 (80.7)	670 (75.5)	1530 (83.2)
Age at circumcision (year)	17.7 (IQR:12–22)	19 (IQR:12–24)	17 (IQR:10–21)

“Donor” refers to respondents who reported ever donating blood, and “Non-donor” refers to those who had never donated blood

### Quantitative findings

#### Attitudes towards blood donation eligibility criteria

A total of 887 respondents (33%) had previously donated blood (Table 2). Overall, 16% (442) of participants supported the current lifetime prohibition against MSM donating blood, while 34% (934) opposed it, and 50% (1349) expressed a neutral attitude. In comparison, 32% (876) of MSM supported hypothetical time-limited deferral policies, 12% (334) opposed them, and 48% (1307) expressed a neutral attitude. An additional 8% (208) reported that they were still unclear about the meaning of deferral policies even after reading the provided explanation.

**Table 2:** Attitudes towards and compliance with blood donation eligibility criteria among men who have sex with men in China

	Total (*N* = 2725)	Donor (*N* = 887)	Non-donor (*N* = 1838)
	*n* (%)	*n* (%)	*n* (%)
Attitudes towards time-limited deferral policy			
Tend to support	876 (32.2)	305 (34.3)	571 (31.1)
Neutral	1307 (48.0)	381 (43.0)	926 (50.4)
Tend to oppose	334 (12.2)	131 (14.8)	203 (11.0)
Still unclear about the meaning	208 (7.6)	70 (7.9)	138 (7.5)
Do you agree with the current prohibition?			
Yes	442 (16.2)	128 (14.4)	314 (17.1)
No	934 (34.3)	397 (44.8)	537 (29.2)
Neutral	1198 (44.0)	315 (35.5)	883 (48.0)
Unsure	151 (5.5)	47 (5.3)	104 (5.7)
Why do you disagree with the current prohibition?*	(*n* = 934)	(*n* = 397)	(*n* = 537)
It is a one-size-fits-all policy	486 (52.0)	218 (54.9)	268 (49.9)
It discriminates against MSM	691 (74.0)	306 (77.1)	385 (71.7)
Blood samples undergo safety testing, so there is no need for a ban	638 (68.3)	281 (70.8)	357 (66.5)
There is a shortage in blood supply, so the ban is unnecessary	294 (31.5)	129 (32.5)	165 (30.7)
Others	26 (2.8)	13 (3.3)	13 (2.4)
Suggestions for changes to the current eligibility criteria*			
Remove the current eligibility criteria and allow all MSM to donate blood	1048 (38.5)	427 (48.1)	621 (33.8)
Those who engage in high-risk sexual behaviors should be prohibited from donating blood, not MSM specifically	1,112 (40.8)	416 (46.9)	696 (37.9)
Implement the time-limited deferral policy	656 (24.1)	230 (25.9)	426 (23.2)
Maintain the current eligibility criteria	771 (28.3)	180 (20.3)	591 (32.2)
Others	37 (1.4)	13 (1.5)	24 (1.3)

^*^These items allowed multiple responses; therefore, percentages may not sum to 100%

In multivariable regression, support for hypothetical time-limited deferral policies (vs non-support) was associated with self-identifying as gay (aPR = 1.15, 95% *CI* 1.00–1.33), being circumcised (aPR = 1.14, 1.01–1.29), a positive attitude towards blood donation (aPR = 1.62, 1.29–2.05) and recognition of the commonly cited rationale that MSM are considered at higher risk for blood-borne infections (aPR = 1.22, 1.09–1.37) (Table 3).

**Table 3:** Attitudes towards time-limited deferral policies and their correlates among men who have sex with men in China, with models adjusted for age, educational level, and income

Characteristic	Support (*n* = 876)		Non-support (*n* = 1641)		Univariable PR (95% *CI*)	Multivariable aPR (95% *CI*)
	*n*	%	*n*	%		
Sexual orientation						
Gay	692	31.8	1236	68.2	1.13 (0.97–1.30)	1.15 (1.00–1.33)*
Bisexual	149	38.9	319	61.1	Ref.	Ref.
Heterosexual	33	29.2	80	70.8	0.92 (0.67–1.26)	0.92 (0.67–1.26)
Others	2	25.0	6	75.0	0.79 (0.23–2.63)	0.82 (0.25–2.65)
Circumcision						
Yes	197	40.4	291	59.6	1.21 (1.07–1.37)*	1.14 (1.01–1.29)*
No	679	33.5	1350	66.5	Ref.	Ref.
Oral intercourse (P1m)						
Yes	366	33.3	734	66.7	Ref.	Ref.
No	493	36.6	854	63.4	1.10 (0.99–1.23)	1.12 (1.00–1.24)*
Unsure	17	24.3	53	75.7	0.73 (0.48–1.11)	0.82 (0.55–1.21)
Attitudes towards blood donation, based on its perceived impact on individuals and society						
Negative/opposed	53	29.9	124	70.1	Ref.	Ref.
Neutral	269	22.7	917	77.3	0.76 (0.59–1.06)	0.79 (0.61–1.01)
Positive/supportive	554	48.0	600	52.0	1.60 (1.27–2.02)*	1.62 (1.29–2.05)*
Have you heard of the commonly stated rationale for MSM blood donation restrictions (i.e., being considered at higher risk for blood-borne infections)?						
Yes	273	43.4	356	56.6	1.36 (1.22–1.52)*	1.22 (1.09–1.37)*
No	603	31.9	1285	68.1	Ref.	Ref.

Attitudes towards time-limited deferral policies were originally assessed on a three-category scale (“support”, “neutral”, “oppose”) and were dichotomized as support versus non-support (neutral/oppose) to facilitate interpretation and to identify factors associated with expressed support. Prevalence ratios (PR) and adjusted prevalence ratios (aPR) were estimated using modified Poisson regression with robust variance, adjusting for age, educational level, and income. P1m indicates behaviors reported during the past 1 month. *CI*: confidence interval. *P < 0.05

#### Willingness to donate blood under hypothetical time-limited deferral policies

**Fig. 2:**
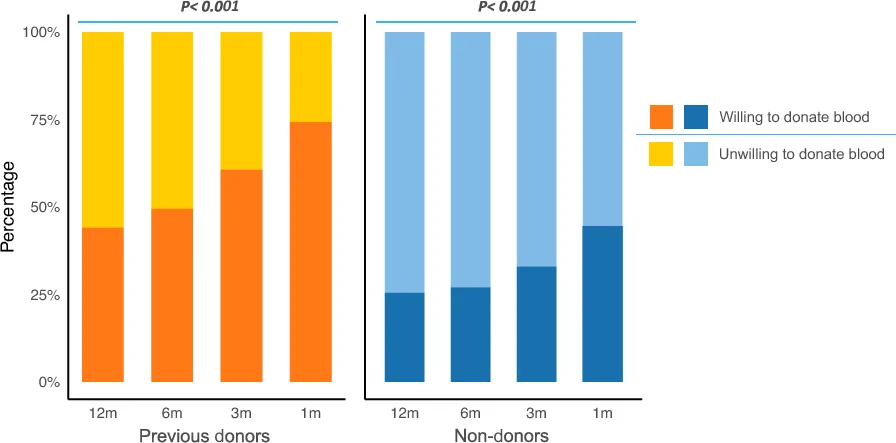
Willingness to donate blood among men who have sex with men under four different hypothetical time-limited deferral policy scenarios between previous donors and non-donors. Previous donors were defined as respondents who reported ever donating blood, and non-donors were defined as respondents who reported never donating blood. The four scenarios correspond to hypothetical deferral periods of 12 months, 6 months, 3 months, and 1 month after the most recent same-sex sexual behavior. Orange bars represent donors, and blue bars represent non-donors. In both panels, darker shades indicate the proportion of individuals willing to donate blood under each hypothetical policy scenario. *P* for trend was estimated using generalized estimating equations accounting for repeated responses from the same participants

In the quantitative survey, willingness to donate was higher under shorter hypothetical deferral periods. As the hypothetical deferral periods shortened from 12 months to 1 month, willingness to donate increased in the overall sample (31%, 34%, 42%, and 54%, respectively; *P* for trend < 0.001). Similar patterns were observed among previous donors (44%, 50%, 61%, 74%; *P* for trend < 0.001) and non-donors (26%, 27%, 33%, 45%; *P* for trend < 0.001) (Fig. 2). Under the current prohibition, among previous donors, the annual donation frequency calculated from self-reported lifetime donation history was 0.20 donations per person-year, with a mean volume of 316 ml per donation. Under the hypothetical deferral scenarios, among respondents willing to donate, reported planned annual donation frequencies ranged from 0.66 to 0.69 donations per person-year, and reported planned mean volumes per donation ranged from 276 to 284 ml. Corresponding values stratified by previous donation history are shown in Supplementary Table S1.

#### Sexual behaviors of MSM willing to donate blood under different hypothetical deferral policies

Table 4 presents the sexual behaviors of MSM who expressed willingness to donate blood under different hypothetical deferral periods. We examined these results in two complementary ways: first, to estimate the proportion of willing donors who would meet the corresponding behavioral criterion. Second, to estimate the proportion of behaviorally eligible MSM who would be willing to donate. Among MSM who expressed willingness to donate under each hypothetical scenario, the proportions reporting no recent anal sex with another man during the corresponding deferral periods were 37% (316) under a 12-month deferral, 42% (391) under a 6-month deferral, 45% (518) under a 3-month deferral, and 53% (792) under a 1-month deferral. Among MSM who reported no anal sex with another man during the past 12-, 6-, 3-, and 1-month periods, the proportions who expressed willingness to donate under the matching hypothetical deferral scenarios were 40% (349), 40% (424), 44% (555), and 53% (829), respectively.

**Table 4:** Willingness to donate and scenario-specific behavioral eligibility under hypothetical time-limited deferral scenarios

Panel A: Among men who have sex with men willing to donate under each scenario, proportion reporting no anal sex during the corresponding period		
Scenario	Willing to donate, *n*	No anal sex during corresponding period, *n* (%)
12-month deferral period	857	316 (36.9)
6-month deferral period	934	391 (41.9)
3-month deferral period	1144	518 (45.3)
1-month deferral period	1475	792 (53.4)

Panel B: Among men who have sex with men reporting no anal sex during the corresponding period, proportion willing to donate under the matching scenario		
Scenario	No anal sex during corresponding period, *n*	Willing to donate,* n* (%)
12-month deferral period	882	349 (39.6%)
6-month deferral period	1055	424 (40.2%)
3-month deferral period	1259	555 (44.1%)
1-month deferral period	1561	829 (53.1%)

### Qualitative findings

A total of 388 survey respondents provided contact information for potential follow-up interviews. Among them, we purposively selected respondents with different blood donation histories, ages, and geographic locations. We attempted to contact 43 respondents; 14 could not be reached, 13 declined participation, and 16 completed qualitative interviews. Interview participants had a mean age of 31 years (range: 25–44 years). They were from 11 different cities (Beijing, Wenzhou, Siping, Hangzhou, Tianjin, Zhuhai, Zhangjiagang, Dehong, Guiyang, Nanning and Qingdao). Seven participants had previously donated blood and nine had not.

The qualitative interviews generated four themes that helped explain participants’ perceptions of hypothetical time-limited deferral policies, including perceived acceptability, feasibility, and views on appropriate deferral length (Table 5).

**Table 5:** Qualitative themes explaining men who have sex with men participants’ perceptions of hypothetical time-limited deferral policies

Theme	Analytic focus
Time-limited deferral was viewed as a more acceptable but still imperfect alternative to the current eligibility criteria	How participants compared hypothetical deferral policies with the current prohibition and interpreted them as incremental but incomplete policy change
Time-limited deferral may increase willingness to donate when participants perceive the requirements as feasible	How perceived feasibility and ability to comply shaped participants’ stated willingness to donate
Preferred deferral length was judged in relation to HIV testing window periods	How participants reasoned about appropriate deferral length using testing window periods, HIV concerns, and abstinence burden
Concerns about the reliability of self-reporting apply to implementation of both lifelong prohibition and time-limited deferral policies	How participants viewed the limits of self-reported sexual behavior and the role of laboratory screening in maintaining blood safety

Themes were derived from semi-structured qualitative interviews with 16 MSM participants. Themes were not mutually exclusive

**Theme 1: Time-limited deferral was viewed as a more acceptable but still imperfect alternative to the current eligibility criteria**

Most of those interviewed (12/16) believed hypothetical time-limited deferral policies were more tolerant and supportive of MSM compared to the current lifelong prohibition. Some participants further suggested that blood donation eligibility criteria should be applied to all individuals who may have engaged in high-risk behaviors, rather than targeting a specific group.

*“Regarding the criteria themself, my personal attitude is that compared to the rigid and outdated prohibition, although the time-limited deferral policy still carries a degree of discrimination, at least it represents some progress, right? And this kind of criteria change may be advanced step by step.”—Donor (age 44, HIV-positive since 2022, last donated blood in 2014, from Tianjin; interview 8)*

*“I think the time-limited deferral policy is definitely a softer approach than the prohibition. But that doesn’t mean it’s perfect—there’s still room for improvement. If there is going to be a deferral period, then it should apply to all individuals who have had sexual activity.”—Non-donor (age 30, HIV-negative, from Hangzhou; interview 11)*

**Theme 2: Time-limited deferral may increase willingness to donate when participants perceive the requirements as feasible**

Many who were interviewed (9/16) said if future eligibility criteria were revised to allow blood donation under a hypothetical time-limited deferral framework, their willingness to donate would increase and they would be willing to comply with such requirements.

*“If such a deferral policy were implemented, it might increase my likelihood of donating blood. After all, since learning about the [current prohibition], I haven’t donated blood for a long time. Also, I haven’t had a sexual partner for several months now, so I could comply with a deferral policy.”—Donor (age 31, HIV-negative, from Hangzhou; interview 13)*

Participants generally opposed deferral periods longer than 1 year, as they believed such policies made it difficult for most people to meet the criteria for safe blood donation and did not represent a substantial improvement over the current prohibition.

*“You mentioned that some countries have implemented deferral periods of 1 year or even 5 years. That’s ridiculous. How many people would remain abstinent from sex for a whole year or 5 years just to donate blood? Rather than such a policy, it would be better to just ban us from donating altogether.”—Donor (age 29, HIV-negative, from Beijing; interview 4)*

**Theme 3: Preferred deferral length was judged in relation to HIV testing window periods**

Most interviewees (13/16) believed the deferral period length should be determined based on the window period of HIV testing technologies currently widely used in blood centers. Some also suggested that individual characteristics and window periods for sexually transmitted infections (STIs) besides HIV should be taken into account.

*“If the deferral period is calculated according to the window period of HIV testing, then it could simply be set at 3 months.”—Donor (age 24, HIV-negative, from Hangzhou; interview 1)*

*“Shouldn’t the deferral period be set according to the sexual behavior patterns of people of different genders and ages across the country? For example, younger individuals may engage in sex more frequently, and an overly long deferral period could place a greater burden on them. Additionally, we should also consider other STIs. When determining the deferral period, we may need to take into account the window periods of various infections and choose an appropriate length.”—Non-donor (age 25, HIV-negative, from Zhuhai; interview 10)*

**Theme 4: Concerns about the reliability of self-reporting apply to implementation of both lifelong prohibition and time-limited deferral policies**

Some interviewees noted that both the current prohibition and hypothetical time-limited deferral policies would rely heavily on self-reported sexual behaviors, raising concerns about verifiability, trust, and practical implementation. Participants emphasized that donor self-disclosure alone may be insufficient to ensure effective screening, and that blood safety ultimately depends on both behavioral screening and laboratory testing.

*“I still think that [deferral policies] cannot truly achieve effective screening in practice.... They still rely on people’s voluntary self-reporting of sexual behaviors. Ultimately, the key to ensuring blood quality lies in the extent to which medical technology can screen for HIV.”—Donor (age 29, HIV-negative, from Beijing; interview 4)*

### Mixed-methods integration

**Fig. 3:**
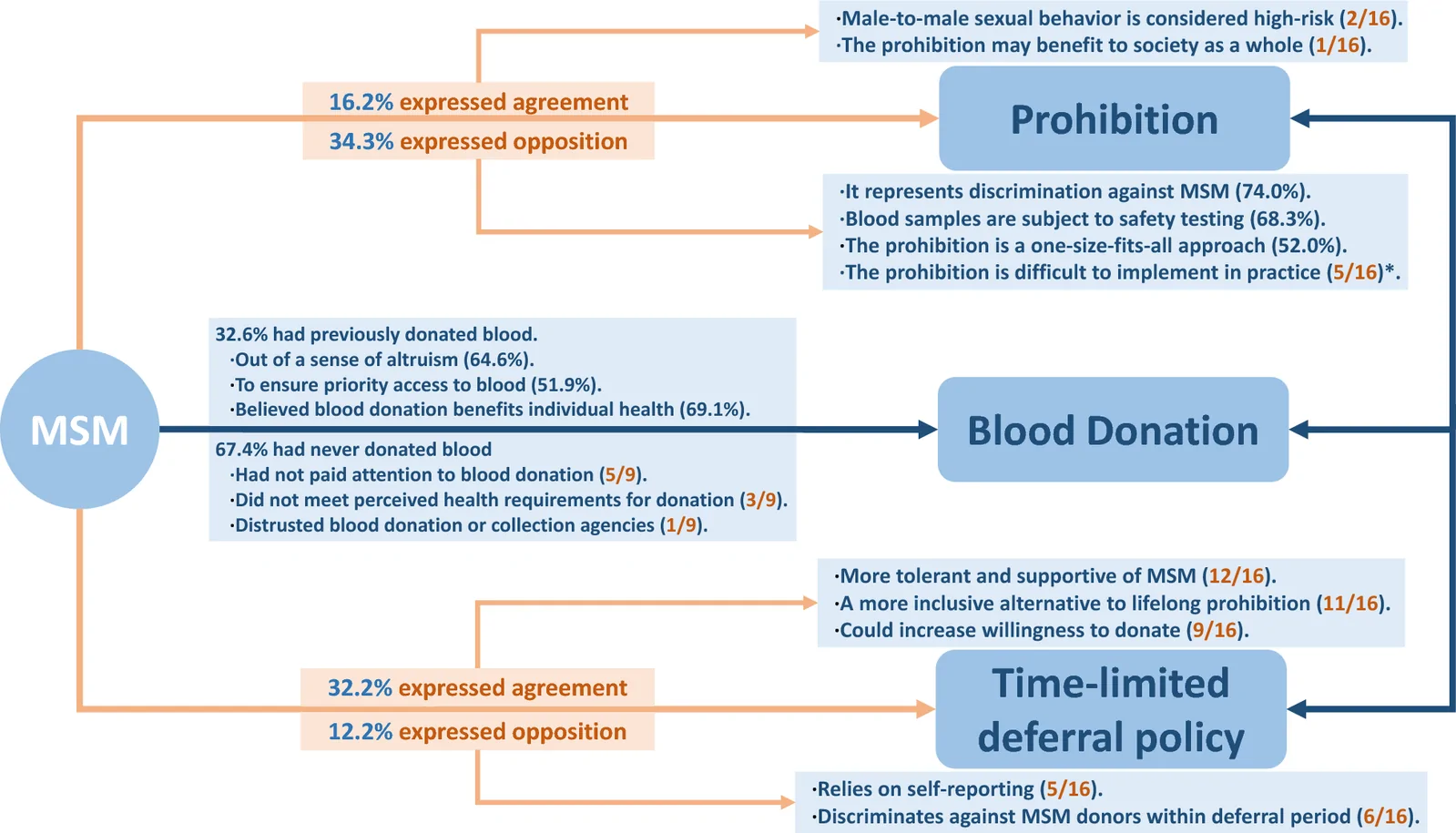
Integrated framework of attitudes, motivations, and compliance related to blood donation eligibility criteria among men who have sex with men. *MSM* men who have sex with men; Prohibition: under the current prohibition, blood donation eligibility criteria exclude populations at higher risk for bloodborne infections, such as MSM or individuals with multiple sexual partners; Time-limited deferral policy, MSM need to defer blood donation for a defined period after their most recent same-sex sexual behavior. Quantitative findings are shown in blue and qualitative findings are shown in orange. Percentages (e.g., “52.0%”) represent quantitative survey findings, and fractions (e.g., “5/16”) represent qualitative interviews findings

Table 6 integrates the quantitative survey findings and qualitative interview themes related to attitudes towards the current prohibition and hypothetical time-limited deferral policies. Figure 3 presents a conceptual framework that links MSM blood donation history and motivations with attitudes towards the current eligibility criteria and hypothetical time-limited deferral scenarios.

**Table 6:** Joint display integrating survey findings and interview themes on attitudes towards the current prohibition and hypothetical time-limited deferral policies among men who have sex with men in China

	*n* (%) (Quantitative)	Quotations (Qualitative)	Mixed methods meta-inferences
Attitudes towards the prohibition against blood donation among MSM			
Agree	442 (16.2)	After all, male-to-male sex is a high-risk behavior, so maybe we really isn’t suitable for blood donation.—*Non-donor (age 45, HIV-negative, from Siping; interview 6)*	Expansion
		If the restriction is reasonable, and it benefits society as a whole, then I think such a policy is acceptable—even if it places limits on a certain group.—*Non-donor (age 23, HIV-negative, from Beijing; interview 3)*	
Disagree	934 (34.3)		
It is a one-size-fits-all policy	486 (52.0)	Even if you think male-to-male sex is high-risk, I don’t think it makes sense to permanently disqualify someone from donating blood just because they’ve done it once.—*Donor (age 24, HIV-negative, from Hangzhou; interview 1)*	Confirmation
		This is a one-size-fits-all policy. It’s essentially discrimination against people with a specific identity.—*Donor (age 29, HIV-negative, from Beijing; interview 4)*	
There is discrimination against MSM	691 (74.0)	A policy like this only causes harm. When people see this kind of policy, it just increases bias against MSM. They’ll assume we’re banned from donating blood because we must be spreading diseases.—*Donor (age 29, HIV-negative, from Beijing; interview 4)*	Expansion
		Is it only MSM who can transmit HIV? Heterosexual people don’t spread it? This policy is extremely discriminatory.—*Non-donor (age 31, HIV-negative, from Hangzhou; interview 7)*	
Blood samples undergo safety testing, so there is no need for a ban	638 (68.3)	There should be more thorough blood screening for people who’ve had recent sexual activity, rather than publicly declaring that MSM can’t donate blood.—*Non-donor (age 25, HIV-negative, from Zhuhai; interview 3)*	Confirmation
		The blood center should be responsible for testing the blood to ensure its safety. We can’t guarantee our bodies are 100% healthy.—*Donor (age 24, HIV-negative, from Hangzhou; interview 1)*	
There is a shortage in blood supply, so the ban is unnecessary	294 (31.5)	There are constant reports about blood shortages, yet the policy bans gay men from donating. That obviously leads to fewer donors—that’s the negative impact of this policy.—*Non-donor (age 29, HIV-negative, from Qingdao; interview 16)*	Confirmation
The implementation of the prohibition is difficult in the real world	Not available in questionnaire	This policy is really just a ‘self-declaration and trust’ system. I could be honest with you, but if I decide to hide the truth, you have no way of verifying it. It completely relies on what the donor says.—*Donor (age 24, HIV-negative, from Hangzhou; interview 1)*	Expansion
		This is a surface-level rule that can’t actually be enforced. There's no way to verify whether donors are telling the truth, so in the end, it just ends up hurting the feelings of the group being targeted.—*Non-donor (age 36, HIV-negative, from Beijing; interview 2)*	
Attitudes towards the time-limited deferral policies			
Agree	876 (32.2)	Compared to the rigid, outdated policy before, this new one is still discriminatory, but at least there’s some change, right? Policy reforms tend to happen step by step.—*Donor (age 44, HIV-positive since 2022, last donated blood in 2014, from Tianjin; interview 8)*	Confirmation
		I would prefer a deferral policy. The outright ban is just too discriminatory. A deferral period feels more tolerant.—*Donor (age 36, HIV-negative, from Wenzhou; interview 9)*	
Disagree	334 (12.2)	This is still a restriction on a particular group, and it might mislead people to associate things that aren’t entirely related. That can also have negative effects.—*Non-donor (age 23, HIV-negative, from Beijing; interview 3)*	Expansion
		I still think that this policy [deferral policies] cannot truly achieve effective screening in practice…. It still relies on people’s voluntary self-reporting of sexual behaviors. Ultimately, the key to ensuring blood quality lies in the extent to which medical technology can screen for HIV.—*Donor (age 29, HIV-negative, from Beijing; interview 4)*	
		A deferral policy probably isn’t ready for implementation in China just yet. Society still isn’t ready to accept behaviors like male-to-male sex. It’ll take broader social progress before people can accept sexual minorities and support this kind of policy reform.—*Donor (age 27, HIV-negative, from Wenzhou; interview 5)*	
The longest acceptable deferral period	Not available in questionnaire–	If the deferral period is based on the HIV window period, then 3 months should be enough. You mentioned some countries used to require 5 years—I honestly can’t imagine anyone giving up sex for 5 years just to donate blood.”—*Non-donor (age 30, HIV-negative, from Hangzhou; interview 11)*	Expansion
		Based on the window period of current testing technology. For example, the fourth-generation HIV antigen/antibody test takes about 3 weeks—then we could extend the deferral period a bit, maybe to 5 weeks.—*Non-donor (age 23, HIV-negative, from Beijing; interview 3)*	
		About a month sounds fine. Blood centers definitely test the blood to control quality anyway, so I don’t think the deferral period needs to be that long.—*Donor (age 27, HIV-negative, from Qingdao; interview 12)*	
The willingness to donate blood under the condition of complying with the time-limited deferral policy	Not available in questionnaire–	If a deferral policy like that were adopted, it might increase the chance I’d donate. Ever since learning about the current prohibition, I haven’t donated. And I haven’t had a partner for a few months, so I could follow the deferral period.—*Donor (age 31, HIV-negative, from Hangzhou; interview 13)*	Expansion
		If a policy like that were in place, and my body index met the requirements of donation, I’d probably go donate—and I’d definitely follow the deferral period.—*Non-donor (age 26, HIV-negative, from Qingdao; interview 15)*

The integrated framework shows that blood donation was relatively common among MSM and was mainly motivated by altruism and anticipated priority access to blood. Support for the current prohibition was limited. Hypothetical time-limited deferral approaches were often viewed as more acceptable and feasible, especially when the deferral period was shorter. Qualitative findings helped explain these patterns by highlighting concerns about practical implementation, reliance on self-reported behaviors, and perceived discrimination. These factors influenced intended adherence under different eligibility approaches.

## Discussion

Our nationwide cross-sectional survey revealed that more than one-third of MSM in China supported hypothetical time-limited deferral policies, and fewer than one-fifth opposed them. Support for hypothetical time-limited deferral policies was associated with sexual orientation, circumcision, positive attitudes towards blood donation, and awareness of the commonly cited rationale for MSM blood donation restrictions. Qualitative findings indicated that many MSM viewed hypothetical time-limited deferral policies as a less discriminatory alternative to the current lifelong prohibition. MSM showed greater willingness to donate under shorter deferral periods. Notably, under the corresponding hypothetical deferral scenarios, nearly half of MSM who reported willingness to donate also reported no anal sex with another man during the specified timeframe, indicating that a substantial proportion would meet the scenario-specific behavioral criteria. Together, these findings provide insight into how MSM assess different blood donation eligibility frameworks under hypothetical conditions and contribute to a better understanding of acceptance and intended behaviors in relation to blood safety considerations.

A key finding of our study is that acceptance of hypothetical time-limited deferral scenarios among MSM varied by deferral length, with shorter deferral periods perceived as more acceptable and feasible than longer ones. Preferences for deferral length may also differ across donor groups. A study conducted in Hong Kong among blood donors from the general population found that respondents more often preferred a longer deferral period for MSM (12 months rather than 1 month) [25]. The authors suggested that this preference may be related to uncertainty or limited awareness of the HIV testing window period among donors [25]. In our study, MSM more often viewed longer hypothetical deferral periods as less acceptable and reported lower intended adherence under these scenarios. Similar challenges related to adherence under long deferral frameworks have also been reported in other settings, including Israel, the United Kingdom, and France [26–28]. These findings suggest that acceptability and intended adherence should be considered when discussing hypothetical deferral scenarios, alongside blood safety requirements. Clear communication of the scientific rationale for eligibility criteria may help improve understanding and support adherence across donor groups.

The correlates of support for hypothetical time-limited deferral policies should be interpreted as indicators of policy perception rather than causal determinants. Participants with more positive attitudes towards blood donation and those who had heard the commonly cited rationale for MSM blood donation restrictions were more likely to support hypothetical deferral policies. These findings suggest that support for time-limited deferral did not simply reflect opposition to blood safety concerns. Instead, some participants may have viewed deferral policies as a more proportionate way to balance donor inclusion, behavioral risk assessment, and blood safety. This interpretation is consistent with the qualitative findings, in which participants often described deferral policies as an imperfect but more acceptable alternative to the current prohibition.

While shorter deferral periods were generally seen more favorably, MSM expressed a range of views towards time-limited deferral scenarios. A key issue raised in the interviews was how eligibility criteria should be defined and implemented in practice. In interviews, some participants expressed concern that criteria based on MSM status or male-to-male sexual behavior as a broad group-level category might be perceived as insufficiently specific or difficult to apply consistently. Several interviewees therefore discussed alternative ways of conceptualizing eligibility criteria, including approaches that focus on recent sexual behaviors at the individual level or that consider the timing of HIV testing. At the same time, participants also noted practical challenges related to self-reported behaviors, including the difficulty of verifying such information during donor screening. These perspectives suggest that acceptability depended not only on the length of the deferral period, but also on whether eligibility criteria were perceived as specific, feasible, consistently applied, and compatible with established blood safety safeguards.

Shorter hypothetical deferral periods were associated with a larger proportion of MSM meeting the scenario-specific behavioral criteria, as fewer individuals reported anal sex with another man during shorter lookback periods. A study by Johns et al. presented similar scenarios (deferral periods of 12 months, 6 months, 2 weeks, and no deferral) [29]. 23% and 37% of respondents qualified under the 12-month and 2-week deferral periods, respectively [29]. Similarly, Romeijn et al. found that 43% of MSM were eligible to donate blood under a minimum deferral period of 4 months [30]. Consistent with these findings, we observed that among MSM willing to donate under the corresponding hypothetical scenarios, 37% and 53% reported no anal sex with another man during the respective 12- and 1-month lookback periods used in the hypothetical deferral scenarios, indicating substantial variation in the proportion meeting scenario-specific behavioral criteria across deferral periods. These behavioral eligibility estimates provide only one part of the evidence needed to evaluate hypothetical deferral approaches. HIV testing window periods and screening technologies remain important contextual factors [7, 9], because behavioral criteria alone are insufficient to determine the blood safety implications of different deferral periods. Therefore, future evaluation of hypothetical deferral approaches may need to assess deferral length together with laboratory screening procedures, the reliability of behavioral information collected during donor screening, and residual blood safety implications within the existing system context.

Our study has several limitations. First, we did not ask participants to separately rate their support for each specific deferral period. Support for time-limited deferral policies was measured as an overall attitude towards the approach. This limited our ability to directly compare perceived acceptability of the 12-, 6-, 3-, and 1-month deferral periods. Second, participants were recruited through sexual health organizations and clinical settings using convenience sampling. Therefore, the sample may overrepresent MSM connected to community organizations or health services and may not be representative of all MSM in China, particularly those in rural areas or less connected to sexual healthcare. Although recruitment was conducted through both online and in-person approaches, recruitment channel was not recorded at the individual level. Therefore, we were unable to compare participant characteristics or responses by recruitment mode. In addition, because the electronic survey platform captured only submitted surveys, unsubmitted incomplete surveys were unavailable, and a conventional response rate could not be calculated. Third, the survey instrument was developed for this study and pretested to improve clarity and acceptability, but it was not formally psychometrically validated. Most items assessed factual information, self-reported behaviors, or single-item attitudes, so internal consistency measures, such as Cronbach’s alpha, could not be used. Fourth, blood donation history and sexual behaviors were self-reported and may be affected by recall or social desirability bias, despite the anonymous design. Fifth, although interviews continued until thematic saturation was reached, some perspectives may not have been captured. Finally, because this was a cross-sectional study based on hypothetical scenarios, we cannot determine how stated willingness would translate into actual donation behavior under future eligibility policies.

## Conclusions

Our study identified a clear trend across hypothetical time-limited deferral scenarios, with willingness to donate increasing as deferral periods shortened. Additionally, under shorter deferral periods, a higher proportion of MSM who were willing to donate blood reported no anal sex with another man during the corresponding timeframe. These findings suggest that shorter hypothetical deferral periods may be associated with a larger pool of potential donors who are both willing to donate and meet the scenario-specific behavioral criteria. Beyond donor eligibility estimates, this study provides China-specific evidence on how MSM perceive different blood donation eligibility approaches, including their acceptability, perceived feasibility, and concerns about implementation. Such evidence may help inform future evaluation of eligibility criteria that aim to maintain blood safety while also considering donor participation, policy acceptability, and perceived fairness. Alternative deferral approaches would need to be evaluated alongside established blood safety measures and within local epidemiological contexts, as behavioral criteria alone cannot determine implications for transfusion safety.

## Supplementary Information

Supplementary material 1: Table S1. Self-reported and planned annual donation frequency and mean volume per donation under the current prohibition and hypothetical time-limited deferral scenarios.

## Data Availability

The datasets used and/or analysed during the current study are available from the corresponding author on reasonable request.

## Abbreviations

aPR: Adjusted prevalence ratio
CDC: Center for Disease Control and Prevention
*CI*: Confidence interval
CNY: Chinese yuan
FPQP: Frozen Plasma Quarantine Programme
GEE: Generalized estimating equation
GVIF: Generalized variance inflation factor
HIV: Human immunodeficiency virus
IQR: Interquartile range
IRB: Institutional Review Board
MSM: Men who have sex with men
P1m: Past 1 month
PR: Prevalence ratio
STI: Sexually transmitted infection
USD: United States dollar
