# Supplementary material for: Time-limited deferral scenarios and blood donation intentions among men who have sex with men in China: a nationwide mixed-methods study
Source: Infect Dis Poverty. 2026 Sep 10;15:101. doi: 10.1186/s40249-026-01496-9 (PMC13560356; doi:10.1186/s40249-026-01496-9)
Supplement: Supplementary file 1 [file 40249_2026_1496_MOESM1_ESM.docx]

Supplementary Table S1. Self-reported and planned annual donation frequency and mean volume per donation under the current prohibition and hypothetical time-limited deferral scenarios.

| **Scenario** | **Overall annual donation frequency, donations/person-year** | **Overall mean volume per donation, ml** | **Previous donors’ annual donation frequency, donations/person-year** | **Previous donors’ mean volume per donation, ml** | **Non-donors’ annual donation frequency, donations/person-year** | **Non-donors’ mean volume per donation, ml** |
| --- | --- | --- | --- | --- | --- | --- |
| Current prohibition | — | — | 0.20 | 316 | — | — |
| 12-month deferral | 0.67 | 284 | 0.66 | 302 | 0.67 | 269 |
| 6-month deferral | 0.68 | 282 | 0.67 | 301 | 0.68 | 266 |
| 3-month deferral | 0.66 | 279 | 0.65 | 298 | 0.67 | 262 |
| 1-month deferral | 0.69 | 276 | 0.73 | 299 | 0.66 | 258 |

Previous donors and non-donors were classified according to self-reported blood donation history at the time of the survey. Under the current prohibition scenario, annual donation frequency and mean volume per donation were calculated only among previous donors based on self-reported lifetime donation history and self-reported usual volume per donation; respondents who had never donated blood did not provide historical donation frequency or donation volume. Under the hypothetical time-limited deferral scenarios, annual donation frequency and volume per donation were based on participants’ reported planned donation frequency and planned volume per donation among those who expressed willingness to donate under the corresponding scenario.
